# Relationship between relevant inflammatory markers and short-term functional outcomes of acute ischemic stroke treated with mechanical thrombectomy: a retrospective cohort study

**DOI:** 10.3389/fneur.2026.1712415

**Published:** 2026-03-12

**Authors:** Jin-Cheng Liu, Jie Wen, Shi-Xiong Peng, Hao Ren, Yiran Liu, Cui Zhou, Xiao Hu, Qi Li

**Affiliations:** 1 Department of Neurology, The Second Affiliated Hospital of Anhui Medical University, Hefei, Anhui, China; 2 Department of Neurology, Xiangyang Hospital of Traditional Chinese Medicine, Xiangyang, Hubei, China

**Keywords:** acute ischemic stroke, functional outcome, inflammation, mechanical thrombectomy, NHR

## Abstract

**Background and purpose:**

Perioperative inflammatory markers are considered critical factors influencing long-term postoperative survival. This study evaluated the neutrophil-to-high-density lipoprotein ratio (NHR), neutrophil-to-lymphocyte ratio (NLR), lymphocyte-to-monocyte ratio (LMR), platelet-to-lymphocyte ratio (PLR), systemic immune-inflammatory response index (SII), and systemic inflammatory response index (SIRI) in relation to functional outcomes in patients with acute ischemic stroke who underwent mechanical thrombectomy (MT). Our objective is to determine the prognostic value of inflammatory composite indices for 90-day functional outcomes in patients with acute ischemic stroke undergoing mechanical thrombectomy and to develop a multivariable prediction model integrating these indices for individualized outcome risk stratification.

**Method:**

A total of 112 patients who underwent MT were enrolled between April 2021 and December 2023. Blood tests were performed at admission. Logistic regression analysis was used to evaluate the relationship between NHR, NLR, PLR, LMR, SII, SIRI and poor functional outcomes at 3 months (mRS Score 3–6). Receiver operating characteristic (ROC) curve analysis was conducted to assess the ability of NHR, NLR, PLR, LMR, SII, and SIRI to predict 90-day functional outcomes.

**Results:**

A total of 54 patients (48%) had poor functional outcomes at 3 months. The median stroke onset to admission time was [6.7] hours (IQR, [3.45]–[8.05]) for poor functional outcome and [9.0] hours (IQR, [7.45]–[14.15]) for good functional outcome. Mean age of the study cohort was 67.5 years, and 64.3% were male. Multivariate logistic regression analysis revealed that NHR (odds ratio [OR], 1.150; 95% confidence interval [CI] 1.002–1.320, *p* = 0.046) was an independent predictor for poor functional outcome after adjusting for other clinical and imaging parameters.

**Conclusion:**

NHR was independently associated with poor functional outcomes at 3 months in patients with acute ischemic stroke who underwent MT. These findings need to be confirmed in larger samples.

## Introduction

Acute ischemic stroke (AIS) results from the sudden occlusion of a cerebral artery, leading to focal cerebral ischemia and rapid neuronal injury (1). AIS represents a principal cause of mortality and long-term disability worldwide, with a persistently increasing global burden in incidence, mortality, and disability-adjusted life years (2). Reperfusion therapy is the cornerstone of AIS management. Intravenous thrombolysis (IVT) with recombinant tissue plasminogen activator (rtPA) administered within a narrow therapeutic window has been shown to significantly improve functional outcomes by restoring cerebral blood flow. However, its efficacy is limited by strict time constraints, contraindications, and reduced effectiveness in large vessel occlusion (LVO) (3). Randomized controlled trials have demonstrated that endovascular therapy is more effective than standard medical therapy for patients with AIS caused by large vessel occlusion (4, 5). Early neurological changes, including early neurological deterioration (END) and early neurological improvement (ENI), are critical short-term markers of clinical trajectory in AIS. END, typically defined as an increase of ≥2–4 points on the National Institutes of Health Stroke Scale (NIHSS) within 24–72 h, occurs in 10–30% of patients and is strongly associated with poor functional outcomes, increased mortality, and higher rates of disability. It often reflects stroke progression, hemorrhagic transformation, cerebral edema, or reperfusion injury. Conversely, ENI—commonly defined as an NIHSS reduction of ≥4–8 points or reaching 0–1 within 24 h—predicts favorable long-term outcomes, including higher rates of functional independence (modified Rankin Scale [mRS] 0–2) at 90 days, particularly after reperfusion therapies such as IVT or mechanical thrombectomy (MT) (6). However, a significant proportion of patients undergoing mechanical thrombectomy experience early neurological deterioration and poor functional outcomes, substantially increasing the burden on patients quality of life (7). Established prognostic factors for AIS outcomes include baseline NIHSS score, age, LVO, infarct volume, time to reperfusion, and comorbidities such as diabetes, atrial fibrillation, and hyperglycemia. Additional markers include imaging features (e.g., Alberta Stroke Program Early CT Score [ASPECTS]), biomarkers (e.g., blood glucose, inflammatory indicators), and reperfusion success (e.g., modified Thrombolysis in Cerebral Infarction [mTICI] grade) (2). Early identification of patients who are at high risk of having poor short-term functional outcomes enables physicians to deliver targeted interventions during hospitalization, potentially reducing the impact of post-stroke complications. Traditional prognostic tools primarily rely on clinical scales and neuroimaging parameters, but these approaches may not fully capture the complex pathophysiological processes—particularly systemic and neuroinflammatory responses—that influence ischemic injury progression and recovery. Therefore, it is essential to utilize rapidly accessible and reliable markers to optimize risk stratification for functional outcomes following MT.

Various researches have revealed that stroke leads to the release of substances such as neuronal cell death products and damage-associated molecular patterns (DAMPs), which activate localized inflammation in the affected brain region (8). It has been shown that peripheral immune cells, particularly neutrophils and monocytes, infiltrate ischemic brain tissue through an impaired blood–brain barrier. Neutrophils contribute to microvascular obstruction, release reactive oxygen species, exacerbating ischemic injury and reperfusion damage. Monocytes/macrophages further modulate tissue injury and repair depending on their polarization state (9–11). In addition, lymphocytes also play a crucial role in the pathophysiology of AIS and post-reperfusion injury. Acute cerebral ischemia induces stroke-related immunodepression, characterized by lymphocyte apoptosis and peripheral lymphopenia, which has been consistently associated with larger infarct volume, increased susceptibility to infections, and worse functional outcomes. There has been a comprehensive meta-analysis including more than 2000 AIS patients found that elevated neutrophil-to-lymphocyte ratio (NLR) was associated with poor functional outcome (OR = 1.55, 95% CI = 1.21–2.00), higher mortality (OR = 2.23, 95% CI = 0.40–13.78), and increased risk of hemorrhagic transformation (OR = 4.32, 95% CI = 2.46–7.61) (12). Although the NLR has been extensively investigated as a prognostic indicator for acute ischemic stroke, the utility of a single NLR as a prognostic indicator in clinical practice is limited. Consequently, it is imperative to explore the combination of multiple inflammatory indicators. Conversely, high-density lipoprotein exhibits anti-inflammatory, antioxidative, and endothelial-protective properties, counteracting vascular injury (13). NHR (neutrophil-to-high-density lipoprotein ratio), a novel inflammatory indicator, can reflect the chronic inflammatory state and lipid metabolism level of the body, and is closely related to the occurrence and development of various cardiovascular and cerebrovascular diseases (14, 15). Compared with traditional ratios such as NLR, NHR integrates both inflammatory cell counts and lipid metabolism status, which may be especially relevant for cerebrovascular diseases where inflammation and atherosclerosis interact to influence stroke progression and post-reperfusion outcomes. Recent studies have demonstrated that elevated NHR is associated with adverse outcomes in patients with acute ischemic stroke, including hemorrhagic transformation and functional prognosis, and provides predictive value independent of conventional risk factors (16). Inflammatory markers, including the NHR, NLR, lymphocyte-to-monocyte ratio (LMR), platelet-to-lymphocyte ratio (PLR), systemic immune-inflammation index (SII), and systemic inflammatory response index (SIRI), have been identified as valuable predictors of functional outcomes in acute ischemic stroke. Previous studies have demonstrated associations between these markers and stroke severity at admission, three-month functional outcomes, and mortality (17–22). Furthermore, a retrospective study found that NLR, PLR, and LMR were correlated with early neurological deterioration after intravenous thrombolysis in patients with AIS (23). However, limited studies have investigated the relationship between inflammatory markers and clinical outcomes in patients undergoing MT after acute ischemic stroke. To date, no study has simultaneously evaluated NLR, PLR, LMR, and NHR as composite inflammatory indices for predicting END and ENI after mechanical thrombectomy.

We aimed to evaluate the predictive efficacy of inflammatory composite indices for short-term functional outcomes in stroke patients following MT and to develop a predictive model using multivariable regression model.

## Methods

### Study participants

Patients with AIS who underwent MT at Xiangyang Traditional Chinese Medicine Hospital between April 2021 and December 2023 were retrospectively screened. Inclusion criteria were as follows: (1) age ≥18 years, (2) acute ischemic stroke within 24 h of symptom onset and underwent MT, (3) MRI performed within 48 h of symptom onset.

Exclusion criteria included pregnancy or breastfeeding, severe cardiac disease (New York Heart Association class III/IV or left ventricular ejection fraction <40%), severe pulmonary disease (oxygen saturation <90%, cyanosis, dyspnea, or abnormal blood gas analyses), severe hepatic dysfunction (serum glutamate aminotransferase >10 times the upper limit of normal), severe renal impairment (serum creatinine >443 μmol/L), active oncological disorders, prior autoimmune disease, steroid use, and active infection at diagnosis. Active infection was defined as the presence of clinical, laboratory, or microbiological evidence of an ongoing infectious process at the time of enrollment, including any of the following: documented diagnosis of acute infection; fever or systemic signs of infection requiring antimicrobial therapy; positive microbiological cultures. This study was approved by the Institutional Review Board of Xiangyang Hospital of Traditional Medicine, and written informed consent was obtained from all patients or their legal representatives.

### Data collection and outcome assessment

Sociodemographic characteristics (age, sex, smoking, and alcohol consumption), medical history (including hypertension, dyslipidemia, stroke history, and other vascular risk factors), laboratory results (including inflammatory parameters and lipid profiles), concurrent medications, clinical information, and imaging characteristics were collected. Large vessel occlusion (LVO) site was confirmed using digital subtraction angiography (DSA) serving as the reference standard in patients undergoing endovascular treatment. All imaging data were independently reviewed by experienced neuroradiologists who were blinded to clinical and laboratory data. Time-to-reperfusion and early neurological improvement, commonly assessed by dynamic changes in NIHSS, are well-established determinants of functional outcome after MT. Stroke severity was assessed using the National Institutes of Health Stroke Scale (NIHSS) at 24 h and 7 days post-intervention. Stroke subtypes were classified according to the TOAST criteria and the Oxfordshire Community Stroke Project (OCSP) classification for partial anterior circulation infarction (PACI). The infarct region was evaluated using the Alberta Stroke Program Early CT Score (ASPECTS), and postoperative modified thrombectomy in Cerebral Infarction (mTICI) grades (0-2a, 2b-3) were documented to assess blood flow. All study participants underwent routine blood tests upon admission to measure neutrophil, lymphocyte, platelet, and monocyte counts. These are presented as absolute values (×10^9^/L). Fasting blood samples at 6 a.m. the next day were collected to assess total cholesterol (TC), triglycerides (TG), low-density lipoprotein cholesterol (LDL-C), and high-density lipoprotein cholesterol (HDL-C). Venous blood was collected upon admission using ethylenediaminetetraacetic acid for anticoagulation. The laboratory standard operating procedures were strictly followed, and the analysis and testing were completed within 2 h to ensure the accuracy of the results. Inflammatory indicators (including NHR = neutrophil/high density lipoprotein, NLR = neutrophil/lymphocyte, PLR = platelet/lymphocyte, LMR = lymphocyte/monocyte, SII = platelet* NLR, and SIRI = monocyte* NLR) were calculated. Short-term functional outcomes were assessed using the modified Rankin Scale (mRS) at 3 months by trained clinicians who were blinded to patients’ laboratory results, including inflammatory markers. Good outcome was defined as mRS 0–2, poor functional outcome was defined as 3–6.

### Statistics analysis

Continuous variables are presented as mean (standard deviation, SD) or median (interquartile range, IQR), while categorical variables are expressed as frequency (%). The Kolmogorov–Smirnov test was used to assess the normality of continuous variables. Comparisons of continuous variables were performed using Student’s *t*-test or the Mann–Whitney *U* test, while categorical variables were analyzed using the chi-square test or Fisher’s exact test, as appropriate. Logistic regression analyses were performed to evaluate the relationships between NHR, NLR, PLR, LMR, SII, SIRI, and clinical outcomes, with results expressed as odds ratios (ORs) and 95% confidence intervals (CIs). To avoid overfitting and reduce bias associated with univariable screening, variable selection was performed using the least absolute shrinkage and selection operator (LASSO) regression with 10-fold cross-validation. The total number of candidate variables entered into the model (*n* = 29), the variables forced into the model based on clinical relevance (age, preoperative NIHSS, and NHR), the value of the optimal penalty parameter selected by cross-validation (*λ*_min = 0.0435), and the number of variables retained after penalization (*n* = 6). We further clarify that λ was selected using the minimum cross-validated error rule, while results obtained using the more conservative 1-SE rule (λ_1se = 0.1102; 4 variables retained) are provided as a sensitivity analysis in the Supplementary material. This two-step approach was chosen to ensure model parsimony while maintaining clinical interpretability, particularly given the limited number of outcome events. Variance inflation factors (VIFs) and tolerance were used to measure multicollinearity. Predictors were removed from the model if they had a VIF > 5. Receiver operating characteristic (ROC) analysis was conducted to assess the overall ability of inflammatory parameters to predict short-term functional outcomes. Internal validation was conducted using bootstrap resampling to obtain optimism-corrected estimates of model discrimination. Continuous variables were centered at their mean values prior to multivariable logistic regression analysis. Categorical variables were entered using dummy coding with clinically relevant reference categories. The full model specification, including the intercept, is reported in Table 1. All statistical analyses were performed using IBM SPSS Statistics (version 26.0) and R-4.5.1.

**Table 1:** Demographics and clinical characteristics.

Variables	Good outcomes *N* = 58	Poor outcomes *N* = 54	*p* value
Demographic characteristics			
Age, years	**66.5 [58, 71]**	**73 [62, 78]**	**0.011**
Sex, male	40 (68.9%)	32 (59.3%)	0.284
Smoking	22 (37.9%)	15 (27.8%)	0.254
Alcohol	17 (29.3%)	12 (22.2%)	0.392
Laboratory data			
TC, mmol/L	4.32 ± 0.99	4.32 ± 1.13	0.991
TG, mmol/L	1.19 [0.80, 1.49]	1.22 [0.86, 1.73]	0.531
HDL-C, mmol/L	1.12 ± 0.25	1.07 ± 0.28	0.297
LDL-C, mmol/L	2.53 ± 0.87	2.56 ± 0.84	0.884
NHR	**5.17 [3.70, 7.79]**	**7.03 [5.18, 9.86]**	**0.002**
NLR	**3.79 [2.14, 8.21]**	**7.27 [3.39, 11.26]**	**0.002**
PLR	**136.63 [89.82, 213.68]**	**171.22 [103.92, 281.77]**	**0.002**
LMR	0.35 [0.19, 0.54]	0.31 [0.22, 0.65]	0.379
SII	**749.06 [470.12, 1,479.33]**	**1,418.89 [695.46, 2,493.12]**	**0.002**
SIRI	**1.90 [0.80, 4.03]**	**2.41 [1.43, 5.66]**	**0.044**
Medical history			
Hypertension	37 (63.2%)	35 (65.5%)	0.800
Hyperlipidemia	13 (22.4%)	11 (20.4%)	0.792
Diabetes	12 (20.7%)	14 (25.9%)	0.512
Previous stroke	14 (24.1%)	15 (27.8%)	0.660
Atrial fibrillation	9 (15.5%)	15 (27.8%)	0.114
Coronary heart disease	16 (27.6%)	17 (31.5%)	0.651
Hypoglycemics	8 (13.8%)	9 (16.7%)	0.672
Antihypertensives	24 (41.4%)	21 (38.9%)	0.788
Antiplatelets	10 (17.2%)	10 (18.5%)	0.860
TOAST classification			
LAA	40 (69.0%)	37 (68.5%)	0.997
CE	16 (27.6%)	15 (27.8%)	
Other etiology	2 (3.45%)	2 (3.70%)	
Vascular occlusion			
Middle cerebral artery	36 (62.1%)	32 (59.3%)	0.509
Internal carotid	12 (20.7%)	12 (22.2%)	
Basilar artery	8 (13.8%)	10 (18.5%)	
Other	2 (3.4%)	0	
Baseline clinical features			
ASPECTS	9 [8, 9]	8 [7.75, 9]	0.549
NIHSS on admission	15.47 ± 5.85	17.63 ± 6.08	0.057
Onset to admission time	**6.7 [3.45, 8.05]**	**9.0 [7.45, 14.15]**	**<0.001**
Intravenous thrombolysis, *n* (%)	**26 (44.8%)**	**11 (20.4%)**	**0.006**
Stent implantation, *n* (%)	54 (93.1%)	49 (90.7%)	0.646
Postoperative clinical features			
Puncture-to-reperfusion time, min	**50.5 [32.25, 70.00]**	**60 [47.25, 110.00]**	**0.007**
24-h NIHSS	**15.49 ± 5.85**	**17.63 ± 5.83**	**<0.001**
7-day NIHSS	**4 [2, 8]**	**16.5 [10, 22]**	**<0.001**
Hemorrhagic transformation	10 (17.2%)	16 (28.6%)	0.121
Antiplatelet therapy	**54 (93.1%)**	**43 (79.6%)**	**0.036**
mTICI 0-2a	2 (3.45%)	6 (11.1%)	0.116
mTICI 2b-3	56 (96.6%)	48 (88.9%)	

Continuous data was expressed as medians (interquartile ranges) or mean (standard deviation), numeric data as counts (percentage).

CE, cardio-embolism; HDL-C, high-density lipoprotein cholesterol; LAA, large-artery atherosclerosis; LDL-C, low-density lipoprotein cholesterol; LMR, lymphocyte-to-monocyte ratio; mRS, modified Rankin Scale; mTICI, modified Thrombolysis in Cerebral Infarction; NHR, neutrophil-to-high-density lipoprotein ratio; NIHSS, National Institutes of Health Stroke Scale; NLR, neutrophil-to-lymphocyte ratio; PLR, platelet-to-lymphocyte ratio; SAO, small-artery occlusion; SII, systemic immune-inflammatory response index; SIRI, systemic inflammatory response index; TC, total cholesterol; TG, triglycerides. Bold values indicate statistical significance (*p* < 0.05).

## Results

### Baseline characteristics

**Figure 1:**
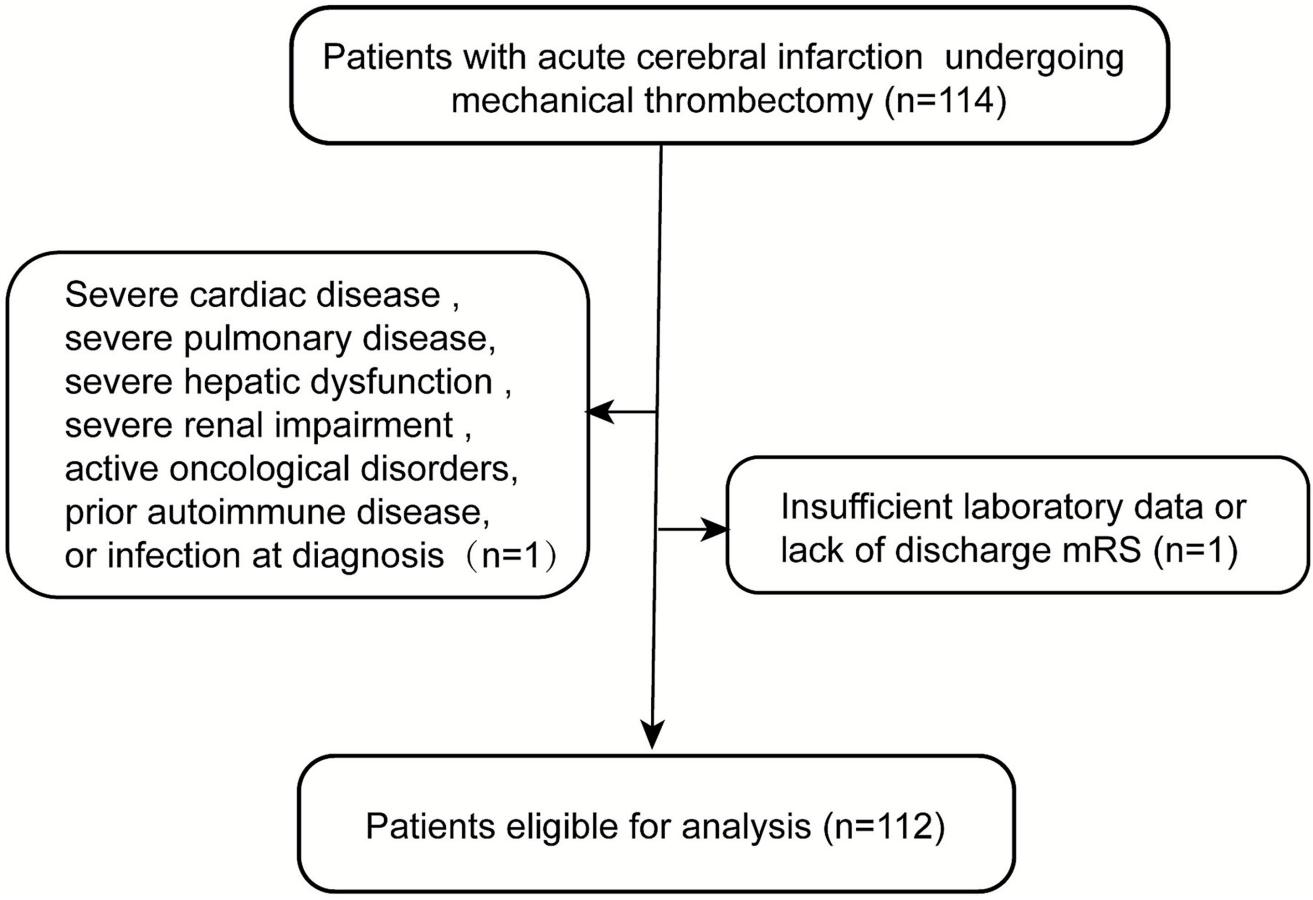
Flow chart of the present study participants.

A total of 112 patients were included (Figure 1), 72 (64.3%) were male. Patients were categorized into a good functional outcome (mRS 0–2) and a poor functional outcome (mRS 3–6) based on the mRS score at 3 months. The baseline characteristics of the participants are presented in Table 2. Participants with worse functional outcomes were older (73 years vs. 66.5 years; *p* = 0.011), had longer recanalization times (60 min vs. 50.5 min; *p* = 0.007), higher NIHSS scores after 24 h (17.62 vs. 15.49; *p* < 0.001), higher NIHSS scores after 7 days (16.5 vs. 4; *p* < 0.001), shorter time of stroke onset to admission time (6.7 vs. 9.0; *p* < 0.001), no intravenous thrombolysis (26 vs. 11; *p* = 0.006) and a lower proportion of antiplatelet medication use (43% vs. 54%, *p* = 0.036).

**Table 2:** Univariable logistic regression analyses for risk factors with poor functional outcome at 3 month.

Variable	OR (95% CI)	P value
Age, years	**1.043 [1.005–1.083]**	**0.027**
Sex, male	0.655 [0.301–1.424]	0.285
Smoking	1.589 [0.716–3.527]	0.255
Alcohol	1.451 [0.617–3.413]	0.393
NHR	**1.171 [1.045–1.313]**	**0.007**
NLR	**1.101 [1.018–1.191]**	**0.016**
PLR	1.002 [0.999–1.006]	0.199
LMR	0.907 [0.779–1.057]	0.213
SII	1.000 [1.000–1.001]	0.055
SIRI	1.003 [0.945–1.063]	0.930
Hypertension	0.956 [0.441–2.073]	0.910
Hyperlipemia	1.129 [0.457–2.792]	0.792
Diabetes	0.745 [0.309–1.797]	0.513
Previous stroke	0.827 [0.355–1.928]	0.661
Atrial fibrillation	0.478 [0.189–1.207]	0.118
Coronary heart disease	0.829 [0.368–1.870]	0.652
Antiplatelets	0.917 [0.348–2.412]	0.860
Large atherosclerotic type		
Cardiac embolism	0.987 [0.428–2.272]	0.975
Other etiological types	1.067 [0.133–8.561]	0.952
ASPECTS	0.914 [0.693–1.206]	0.526
NIHSS on admission	1.063 [0.997–1.134]	0.060
Intravenous thrombolysis	**3.176 [1.371–7.360]**	**0.007**
Stent implantation	1.378 [0.350–5.424]	0.647
Puncture-to-reperfusion time	**1.014 [1.003–1.025]**	**0.016**
24-h NIHSS after operation	**1.193 [1.110–1.283]**	**<0.001**
7-day NIHSS after operation	**1.254 [1.156–1.361]**	**<0.001**
Antiplatelet therapy	**3.453 [1.027–11.610]**	**0.045**
Hemorrhagic transformation	0.495 [0.202–1.214]	0.124
Postoperative mTICI	3.500 [0.675–18.153]	0.136

ASPECTS, Alberta Stroke Program Early CT Score; HDL-C, high-density lipoprotein cholesterol; LDL-C, low-density lipoprotein cholesterol; LMR, lymphocyte-to-monocyte ratio; NIHSS, National Institutes of Health Stroke Scale; NHR, neutrophil-to-high-density lipoprotein ratio; NLR, neutrophil-to-lymphocyte ratio; PLR, platelet-to-lymphocyte ratio; SII, systemic immune-inflammatory response index; SIRI, systemic inflammatory response index; TC, total cholesterol; TG, triglycerides; mTICI, modified Thrombolysis in Cerebral Infarction. Bold values indicate statistical significance (*p* < 0.05).

### Inflammatory indicators and functional outcomes

Patients in the poor functional outcome group exhibited significantly higher inflammatory markers, including NHR (7.03 vs. 5.17; *p* = 0.002), NLR (7.27 vs. 3.79; *p* = 0.002), PLR (171.22 vs. 136.63; *p* = 0.002), SII (1,418.89 vs. 749.06; *p* = 0.002), and SIRI (2.41 vs. 1.90; *p* = 0.044) (Table 2).

**Figure 2:**
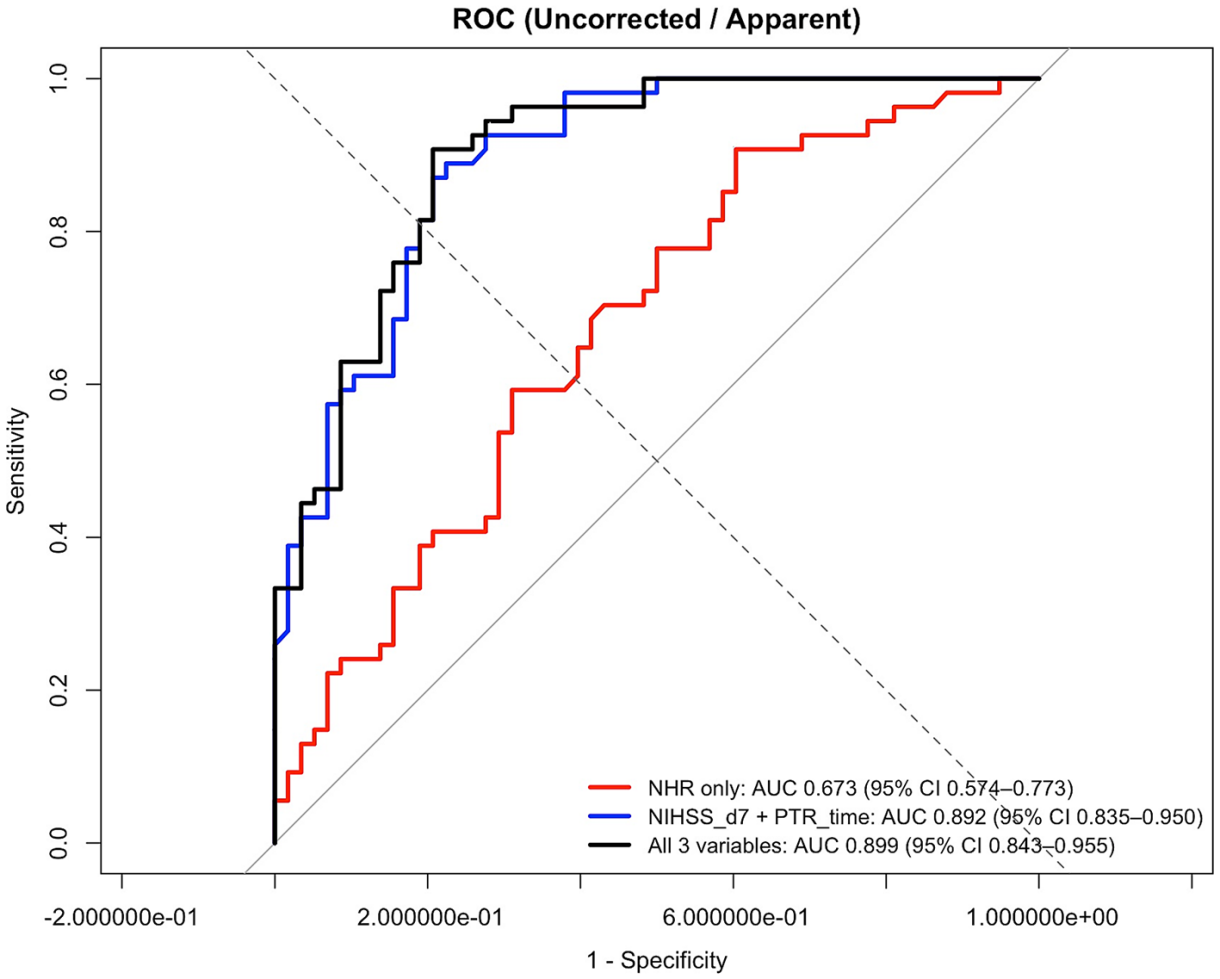
Receiver operating characteristic curve of NHR. The AUC was 0.673 (0.574–0.773) in red line for NHR. The AUC was 0.892 (0.835–0.950) in blue line for the combined prediction of 7-day NIHSS after operation and puncture-to-reperfusion time. The AUC was 0.899 (0.843–0.955) in black line for the full multivariable model.

**Figure 3:**
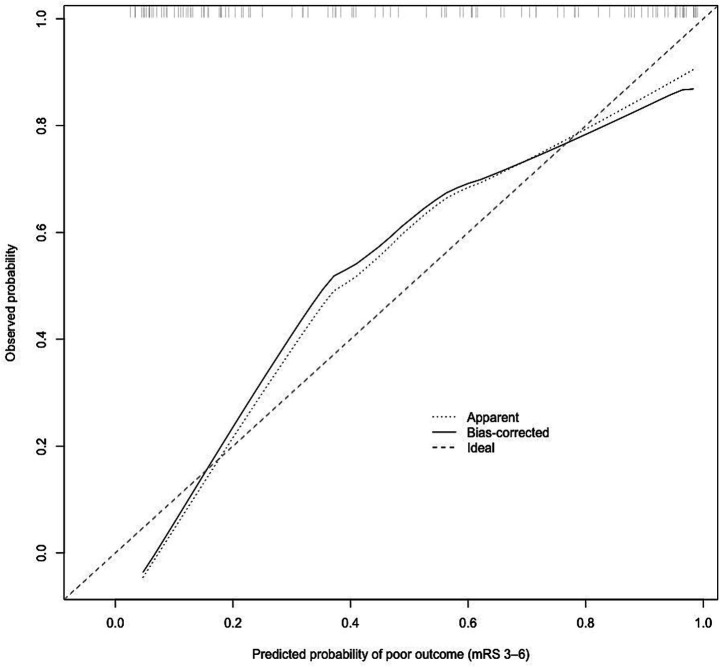
Predicted probabilities were plotted against observed event rates across deciles of predicted risk. The dashed line represents perfect calibration.

Table 3 shows the results of univariable logistic regression analyses for presenting the prognosis of dysfunction. The univariable logistic regression analysis showed that age, time from thrombus removal to recanalization, NIHSS scores at 24 h and 7 days postoperatively, NLR, NHR, intravenous thrombolysis and postoperative use of antiplatelet aggregation medications were associated with poor functional prognosis (*p* < 0.05). In the multivariable analysis (Table 1), after adjusting for ages, ASPECT score, NIHSS score after 7 days, interval between thrombectomy and recanalization, and postoperative use of antiplatelet aggregation medications, NHR values (1.150 [1.002–1.320], *p* = 0.046) was independently associated with poor outcomes. After variable selection using LASSO regression, NHR, age, preoperative NIHSS score, and 7-day postoperative NIHSS score were included in the final multivariable logistic regression model (Supplementary Table 2). Higher NHR was independently associated with poor functional outcome (mRS score 3–6) (OR = 1.165, 95% CI 1.024–1.360, *p* = 0.030). In addition, higher NIHSS score at 7 days after mechanical thrombectomy was a strong predictor of poor outcome (OR = 1.311, 95% CI 1.188–1.483, *p* < 0.001). Preoperative NIHSS score was also independently associated with outcome (OR = 0.885, 95% CI 0.783–0.983, *p* = 0.033), whereas age was not significantly associated with poor functional outcome in the adjusted model (*p* = 0.731). Table 4 was adjusted in the same way as Table 3, but NLR (1.024 [0.940–1.116], *p* = 0.581) is not an independent factor for poor outcomes. ROC curves were generated to evaluate the predictive value of NHR on functional prognosis in patients undergoing MT, as illustrated in Figure 2. The area under the curve (AUC) in blue line for NHR was 0.673 (95% CI, 0.574–0.773). The optimal diagnostic cutoff for NHR was determined to be 4.563, yielding a sensitivity of 90.7% and a specificity of 39.7%. The AUC was 0.892 (0.835–0.950) in blue line for the combined prediction of 7-day NIHSS after operation and puncture-to-reperfusion time. The AUC was 0.899 (0.843–0.955) in black line for the full multivariable model. Calibration assessment showed good agreement between predicted and observed risks (Figure 3). The predictive performance of the multivariable logistic regression model was further evaluated. The model demonstrated good discrimination, with an apparent C-statistic of 0.899. After internal validation using bootstrap resampling (1,000 iterations), the optimism-corrected C-statistic was 0.890 (Supplementary Table 1). Using LASSO regression (Supplementary Table 2 and Table 3), the number of candidate predictors was reduced from 29 to 6, and then further to 4 variables with non-zero coefficients. Calibration assessment showed good agreement between predicted and observed risks (Figure 3).

**Table 3:** Multivariable logistic regression models for poor function outcome at 3 month.

Variable	Poor functional outcome (mRS score 3–6)	
	OR (95% CI)	*p* values
NHR = neutrophil/high-density lipoprotein	**1.150 [1.002–1.320]**	**0.046**
Age	1.016 [0.962–1.071]	0.563
ASPECT score	1.111 [0.734–1.682]	0.619
Cerebral infarction subtype of OCSP	0.505 [0.185–1.382]	0.183
Postoperative antiplatelet therapy	0.520 [0.153–1.770]	0.295
24-h NIHSS after operation	0.990 [0.870–1.126]	0.874
7-day NIHSS after operation	**1.263 [1.109–1.440]**	**<0.001**
Puncture-to-reperfusion time	**1.018 [1.004–1.032]**	**0.012**

Continuous variables were mean-centered before model fitting. Reference categories were as follows: low NHR, Partial anterior circulation infarction, and no postoperative antiplatelet therapy. The model intercept was −5.643. Bold values indicate statistical significance (*p* < 0.05).

**Table 4:** Multivariable logistic regression models for poor function outcome at 3 month.

Variable	Poor functional outcome (mRS score 3–6)	
	OR (95% CI)	P values
NLR = neutrophil/lymphocyte	1.024 [0.940–1.116]	0.581
Age	1.009 [0.959–1.062]	0.725
ASPECT score	1.100 [0.735–1.645]	0.644
Cerebral infarction subtype of OCSP	0.591 [0.219–1.599]	0.300
Postoperative antiplatelet therapy	0.607 [0.188–1.962]	0.404
24-h NIHSS after operation	0.994 [0.874–1.129]	0.922
7-day NIHSS after operation	**1.263 [1.113–1.434]**	**<0.001**
Puncture-to-Reperfusion time	**1.017 [1.004–1.031]**	**0.013**

NHR, neutrophil to high density lipoprotein ratio; NLR, neutrophil-to-lymphocyte ratio; NIHSS, National Institutes of Health Stroke Scale. Bold values indicate statistical significance (*p* < 0.05).

## Discussion

In the present research, we investigated the associations between selected inflammatory biomarkers with functional outcome at 3 months in patients with AIS who underwent MT. Multivariable logistic regression analysis identified NHR as an independent predictor of poor functional outcomes after adjusting for confounding factors. To further evaluate the discriminative ability of inflammatory indices for predicting short-term functional outcomes after MT, ROC curve analysis was performed. NHR demonstrated a moderate predictive performance, with an AUC of 0.673 (95% CI: 0.574–0.773). While this level of discrimination is insufficient for standalone clinical decision-making, it suggests that NHR may serve as a useful adjunctive biomarker reflecting systemic inflammatory status. At the optimal cut-off value, NHR yielded a sensitivity of 90.7% and a specificity of 39.7%, suggesting a reasonable balance between sensitivity and specificity in clinical risk stratification. These findings underscore the clinical value of NHR in early identification of poor outcomes in ischemic stroke patients undergoing MT, highlighting the association between inflammatory activation and its potential impact on stroke prognosis. In addition, it should be noted specifically that the observed association between inflammatory markers and short-term outcomes should be interpreted in the context of reperfusion timing.

Several well-established clinical predictors of functional outcome after mechanical thrombectomy, including NIHSS score at day 7 and puncture-to-reperfusion time, deserve particular attention. Previous studies have consistently demonstrated that early neurological status, especially NIHSS assessed within the first week after stroke, is a strong surrogate marker of final functional outcome (24). Similarly, puncture-to-reperfusion time has been widely recognized as a critical determinant of prognosis, reflecting both procedural efficiency and the extent of ischemic injury. Shorter reperfusion times are associated with higher rates of favorable functional outcomes and reduced infarct progression (25). In this context, inflammatory markers may exert their effects through multiple mechanisms, including modulation of reperfusion injury, blood–brain barrier disruption, and post-ischemic neuroinflammation. Elevated systemic inflammation has been associated with poorer early neurological recovery, which may partly explain its relationship with short-term functional outcomes. Neutrophils, in particular, play a role in the various stages of atherosclerosis. They promote the formation of unstable atherosclerotic plaques by exacerbating endothelial dysfunction, inducing monocytes, activating macrophages, and promoting foam cell formation (26–28). This can lead to complications such as plaque rupture, bleeding, or thrombosis (29). Cerebral ischemia leads to extensive cellular stress and necrosis, resulting in the release of DAMPs. These endogenous danger signals activate pattern recognition receptors, thereby initiating a robust innate immune response. Following DAMP-mediated immune activation, circulating leukocytes, particularly neutrophils and monocytes, are rapidly recruited to the ischemic brain (30). Although reperfusion accomplished via MT is essential for tissue rescue, in situations where an excessive inflammatory response occurs, it may also aggravate the damage to the blood–brain barrier. Blood–brain barrier breakdown facilitates leukocyte infiltration and cerebral edema, and has been implicated in hemorrhagic transformation and poor neurological recovery. Therefore, elevated systemic inflammatory markers may serve as indicators of damage to the blood–brain barrier after reperfusion (31, 32). Oxidative stress represents another key mechanism linking inflammation to secondary brain injury after ischemic stroke. Reperfusion promotes excessive production of reactive oxygen and nitrogen species, particularly from activated neutrophils and damaged mitochondria. These free radicals trigger lipid peroxidation, DNA damage, and neuronal apoptosis, thereby exacerbating ischemic injury despite successful recanalization (33). All in all, sudden interruption of cerebral blood flow leads to neuronal death, resulting in compromised brain cells that produce large quantities of inflammatory cytokines, chemokines, reactive oxygen species, and other neurotoxic substances. These factors contribute to the disruption of the blood–brain barrier and initiate an inflammatory cascade. Additionally, they attract immune-inflammatory cells into the brain tissue, further mediating secondary neuronal damage and exacerbating neurological dysfunction (34). On the contrary, the reconstruction and termination of the inflammatory process is related to lymphocyte function. An increasing number of studies indicate a link between low lymphocyte counts, larger infarct areas and poorer neurological outcomes (35–37). Early research also showed a significant association between stroke severity and the number of leukocytes in the peripheral bloodstream (38). The phenomenon is more noticeable in AIS combined with large-vessel occlusion (39). In addition, neutrophil markers were determined in human carotid atherosclerosis specimens, reinforcing support for their relevance in plaque formation (40). Neutrophils are the earliest blood immune cells to invade ischemic brain tissue (41). Recently, novel biomarkers, such as NHR, have shown promise in predicting inflammatory processes beyond traditional markers. While NLR and PLR mainly reflect systemic inflammatory burden, NHR integrates both inflammatory response and lipid metabolism, which may provide additional prognostic information in ischemic stroke patients. HDL cholesterol plays crucial cardioprotective roles in stroke pathogenesis, including reverse cholesterol transport, anti-inflammatory properties, and enhanced endothelial function. Low HDL levels are associated with an increased risk of ischemic stroke, potentially due to their proatherogenic and antithrombotic properties (42). This suggests that elevated levels of HDL cholesterol may decrease the risk of poor prognosis in patients with atherosclerotic stroke (43). Zhang et al. demonstrated that the neutrophil-to-HDL ratio is a reliable and sensitive independent predictor of bleeding conversion in patients with acute ischemic stroke (16). It has also been reported that elevated NHR levels are associated with a poor short-term prognosis following intravenous thrombolysis in patients with acute ischemic stroke. Chen et al. retrospectively sought that patients with higher NHR levels had higher rates of NIHSS admission, 24-h NIHSS, 7-day NIHSS, and 3-month mRS, as well as higher rates of neutrophil count and hyperlipidemia, which is consistent with our results (43). Importantly, the high discriminative performance observed in the full multivariable model reflects the combined contribution of inflammatory markers and key clinical variables, rather than the predictive capacity of any single biomarker. This finding is also consistent with prior evidence indicating that multivariable models integrating clinical severity, neurological dynamics, and biological markers outperform isolated predictors in stroke outcome prediction. Therefore, NHR might serve as a promising predictor for risk stratification in AIS patients underwent MT. In addition, we acknowledge that previous studies have reported NLR as an independent predictor of poor functional outcomes in AIS. However, our study population differed substantially from previous reports, since we only included patients with large vessel occlusion who were treated with MT. Second, NHR incorporates high-density lipoprotein cholesterol, which possesses anti-inflammatory, antioxidant, and endothelial-protective properties. Therefore, NHR may better reflect the complex interaction between inflammation, oxidative stress, and vascular injury in the setting of AIS treated with MT.

It is noted that relying solely on variation of current routine blood test may not be sufficient to fully understand the complexity of the immune state and response. Thus, the utilization of indices and ratios that combine multiple cellular measurements may provide a more reliable measure for medical treatment purposes. NHR, NLR, PLR, LMR, SII and SIRI are representatives of various combinations of inflammatory parameters that may provide more information about immune activity in the pathogenesis of ischemic stroke and provide more help in the early prognosis of patients with mechanical retrieval of thrombus. What’s more, these six complex markers of inflammation can be calculated from blood cell counts, hence they are comparatively easier to acquire.

Our study has a few limitations. Firstly, this study is a single-center cohort with a small sample size. Patient selection, treatment protocols, and peri-procedural management may vary across institutions, potentially limiting the generalizability of the results. For example, the predictive ability of NHR on functional prognosis in patients undergoing MT is rather suboptimal for clinical utility. Despite the use of methods aimed at reducing overfitting, including LASSO and bootstrap validation, the stability of the predictive model remains limited due to the small number of outcome events, which warrants caution in interpreting the reported discrimination metrics. Secondly, only short-term outcomes were evaluated. The changes in NHR, a dynamic indicator, may be closely related to prognosis. Lastly, imaging parameters were not fully incorporated into the predictive model. Future studies will expand the sample size and conduct multi-center collaborative studies to validate the proposed predictive model, while focusing on predicting long-term prognoses in patients undergoing MT.

However, the successful distribution of recanalization (e.g., the mTICI 2b-3 rate) is closely related to prognosis (44). Previous clinical trials have demonstrated that EVT achieves TICI 2b-3 recanalization in over 85% of AIS patients (45). Even if the reinfusion is successful, 48.7% of AIS patients will experience adverse outcomes (46). Neuroinflammation and imaging parameters are also closely related. Research results have shown that NLR is an independent predictor of poor clinical outcomes after EVT (44). Therefore, identifying patients at risk of adverse outcomes such as ineffective reperfusion and neuroinflammation may help to ensure they receive timely treatment, which could be a promising research direction for future. Due to the limitations of assessing only short-term results, we will include long-term follow-up and quality of life assessments in future studies. Additionally, future researches require a larger sample size and multicenter collaboration to develop an effective tool for predicting the prognosis of MT patients. Importantly, given the retrospective design and the limitations inherent to the available data, our results are not intended to support immediate clinical implementation or to provide a ready-to-use clinical decision-making algorithm. Rather, the primary value of this study lies in highlighting potential directions for future research and in supporting the need for prospective, well-designed studies to validate the clinical utility of markers such as the NHR.

## Conclusion

NHR is associated with short-term functional outcomes of acute ischemic stroke with mechanical thrombectomy, incorporating this parameter into clinical practice may improve risk stratification in IS patients undergo MT.

## Data Availability

The original contributions presented in the study are included in the article/Supplementary material, further inquiries can be directed to the corresponding author.
