# Supplementary material for: Relationship between relevant inflammatory markers and short-term functional outcomes of acute ischemic stroke treated with mechanical thrombectomy: a retrospective cohort study
Source: Front Neurol. 2026 Mar 12;17:1712415. doi: 10.3389/fneur.2026.1712415 (PMC13017336; doi:10.3389/fneur.2026.1712415)
Supplement: Supplementary file 1 [file Table_1.DOC]

**Supplementary Material**

**Supplement Table 1. Discriminative Performance of the Multivariable Logistic Regression Model Based on Table 3**

| Metric | **Estimate** |
| --- | --- |
| Apparent C-statistic | 0.899 |
| Optimism-corrected C-statistic (bootstrap) | 0.890 |
| Bootstrap resamples (n) | 1000 |
| Abbreviations: NIHSS = National Institutes of Health Stroke Scale.  Note: Discrimination was evaluated using the C-statistic. Internal validation was performed with bootstrap resampling (1,000 iterations) to obtain optimism-corrected estimates. The model included NHR, 7-day post-procedural NIHSS score, and puncture-to-reperfusion time. | |

**Supplement Table 2.** **Multivariable Logistic Regression Analysis of Poor Functional Outcome With LASSO (lamda_min) Variable Selection**

| Variable | **Poor Functional Outcome**  **(mRS Score 3-6)** | |
| --- | --- | --- |
|  | OR (95% CI) | P values |
| **NHR** | **1.209 [1.049-1.437]** | **0.015** |
| Age | 0.995 [0.942-1.052] | 0.858 |
| **NIHSS preoperative** | **0.861 [0.747-0.970]** | **0.024** |
| **7-day NIHSS after operation** | **1.336 [1.199-1.539]** | **<0.001** |
| **Puncture to Reperfusion tiome** | **1.014 [1.001-1.028]** | **0.039** |
| **Antiplate therapy postoperation1** | **0.159 [0.022-0.925]** | **0.049** |
| Abbreviations: CI = confidence interval; LASSO = least absolute shrinkage and selection operator; mRS = modified Rankin Scale; NHR = neutrophil-to-high-density lipoprotein ratio; NIHSS = National Institutes of Health Stroke Scale; OR = odds ratio. Lamda_min = 0.0435. | | |

**Supplement Table 3.** **Multivariable Logistic Regression Analysis of Poor Functional Outcome With LASSO (lamda 1se) Variable Selection**

| Variable | **Poor Functional Outcome**  **(mRS Score 3-6)** | |
| --- | --- | --- |
|  | OR (95% CI) | P values |
| **NHR** | **1.165 [1.024-1.360]** | **0.030** |
| Age | 1.009 [0.960-1.062] | 0.731 |
| **NIHSS preoperative** | **0.885 [0.783-0.983]** | **0.033** |
| **7-day NIHSS after operation** | **1.311 [1.188-1.483]** | **<0.001** |
| Abbreviations: CI = confidence interval; LASSO = least absolute shrinkage and selection operator; mRS = modified Rankin Scale; NHR = neutrophil-to-high-density lipoprotein ratio; NIHSS = National Institutes of Health Stroke Scale; OR = odds ratio. Lamda 1se = 0.1102 | | |
